# Patients might distrust honest doctors

**DOI:** 10.1002/jhm.70416

**Published:** 2026-07-28

**Authors:** Byron Hyde

**Affiliations:** ^1^ Department of Population Health Sciences Bristol Medical School Bristol UK; ^2^ School of History, Law and Social Sciences Bangor University Gwynedd Wales UK

## Abstract

Evidence‐based medicine revolutionized clinical practice, bringing expectations that physician–patient communication should be similarly transparent about scientific evidence and uncertainty. However, the replication crisis has revealed significant weaknesses in biomedical research. This creates something of a paradox: honest disclosure of uncertainty is ethically obligatory but often breeds patient distrust rather than confidence. This commentary argues that trust in medicine requires systemic educational reform to improve public scientific literacy, and offers some practical communicative strategies physicians can employ immediately, including deliberate language hedging and setting realistic expectations. Critically, such approaches only succeed when grounded in compassionate physician–patient relationships. In an era of increasing commercialization and artificial intelligence in medicine, preserving this fundamental relationship is essential for maintaining public trust in healthcare.

## Visual Abstract

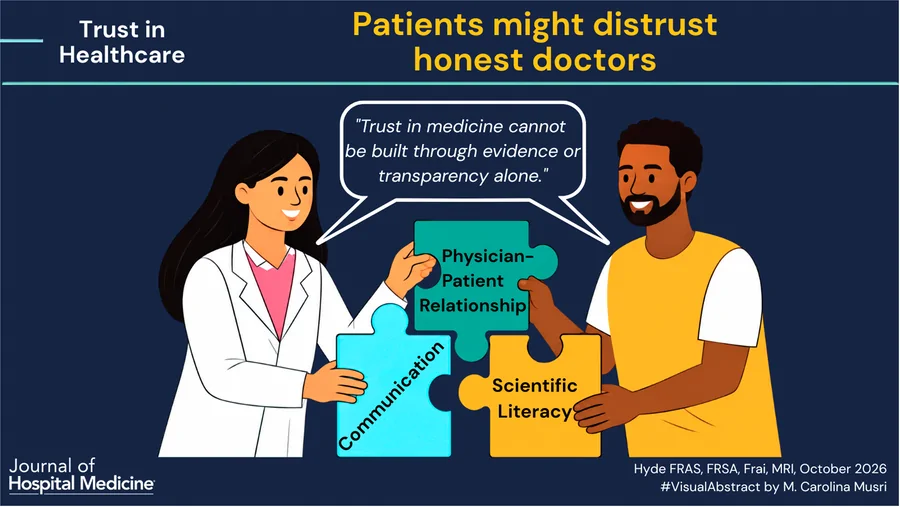

Around 30 years ago, evidence‐based medicine was finding its feet.^1^ The movement promised to revolutionize healthcare by grounding clinical decisions in rigorous scientific evidence rather than tradition or intuition. Alongside this came a parallel commitment: that our communication with patients should be evidence‐based too.^2^, ^3^ We'd tell patients what the evidence actually showed—no more, no less. It sounded like progress.

But just as evidence‐based medicine was gaining momentum, biomedical science faced a reckoning. The replication crisis revealed that much of what we'd taken as established knowledge couldn't be reliably reproduced.^4^, ^5^ In cancer research, only about 40% of studies could be replicated.^6^, ^7^ Some philosophers of medicine went so far as to argue we can't trust medical science at all.^8^ If experts are questioning the foundations of medicine, what's a practicing physician supposed to tell their patients?

Here's the problem: honest disclosure of uncertainty breeds distrust.^9^ When we're transparent about what we don't know or what might go wrong, patients often interpret that honesty as incompetence rather than integrity. They might lose trust in their individual physician, or in medicine as a whole—the empirical literature is currently uncertain about the relationship between the objects of patient trust. Consider conflict of interest disclosures. You'd think transparency would improve trust, but the evidence suggests otherwise. The Physician Payments Sunshine Act increased transparency around physicians’ financial conflicts of interest across the United States.^10^ The result: patients lost trust in their physicians, even if they hadn't received any payments.^11^ More recently, when journals such as Nature and the New England Journal of Medicine endorsed the Democratic candidate for President of the United States, the public lost trust in science as a whole, not just in those journals.^12^ Scholars are increasingly becoming aware of a disconnect between trust and trustworthiness, and of circumstances where increases in the *trustworthiness of* science and medicine have detrimental effects on public *trust in* them.^13^, ^14^, ^15^, ^16^, ^17^

So should doctors simply withhold information? Should we return to a paternalistic model where physicians made decisions and patients simply complied? That seems obviously wrong, even if it might be pragmatically effective in some narrow sense. Patients have a fundamental right to an explanation of their care and the reasoning behind clinical decisions,^18^ and physicians have a legal duty of candour in most jurisdictions.^19^, ^20^, ^21^ We can't simply decide that patients are better off kept in the dark because they can't handle the messy reality of medical uncertainty. That would be a profound betrayal of the principles that should guide modern medicine.

Paradoxically, there's also evidence pointing in the opposite direction. Some studies suggest that honest communication about uncertainty *increases* public trust and protects against erosion of confidence when evidence changes and health advice shifts accordingly.^22^ It's also been found that some patients interpret conflicts of interest as markers of expertise, leading them to trust conflicted physicians *more*, not less.^23^, ^24^ What explains this so‐called “transparency paradox” is what we're honest about. When physicians disclose bad news—conflicts of interest, uncertainty, misconduct, and so on—the public loses trust in medicine; when they disclose good news—strong statistical inferences, “clean” (nonindustry) funding, and so on—then the public trusts them more.^25^

How can physicians escape this paradox, whereby attempts to increase trust simultaneously risk losing it, without simply lying about the bad news? The underlying solution lies beyond medicine itself: the public needs to understand science better.^26^ What's required isn't just more education in the sense of more years in school or more science classes, but a different kind of education about how scientific knowledge develops, evolves, and occasionally gets revised or even overturned.^27^, ^28^ Patients should understand how science works, not just what science says; that uncertainty isn't a bug in the system but a feature of honest science. That's a lesson for governments and education policymakers. Patients certainly can't be blamed for not perusing the latest evidence‐based medicine literature. But public education won't change overnight (and might not change at any point soon). In the meantime, what can physicians actually do in the clinic today?

In the absence of work by educational institutions, the institution of medicine has historically assumed responsibility for bridging the gap in understanding through tools like plain language summaries. I suggest that the patient–physician relationship itself can be a vehicle for scientific literacy. We can teach our patients how science actually works through our interactions with them.^29^ This doesn't mean turning every consultation into a philosophy of science seminar or lecturing patients about probabilistic reasoning. It can be remarkably simple and woven naturally into everyday clinical conversations with patients.

Physicians might consider hedging their language just slightly. Instead of presenting a treatment as definitive, try something like: “I expect this will work because the evidence suggests it should. But this is a relatively new drug, so there's a chance it won't be as successful as we're hoping. If that happens, we'll try…” This simple reframing accomplishes several things simultaneously: it satisfies physicians’ duty of candour and respects patients’ right to an explanation; it signals expertise—you know the literature and understand the drug's limitations; and it normalizes scientific uncertainty, sets realistic expectations, and makes your proposed treatment misunderstanding‐proof. If the treatment doesn't work as hoped, the patient is less likely to conclude that the physician is incompetent and lose trust in them.

However, these communication strategies only work if they're grounded in something deeper: compassionate, genuinely human interactions with patients.^30^ This prevents patients from hearing explanations about scientific limitations merely as excuses for incompetence. Patients must believe that their physicians genuinely have their best interests at heart. The evidence suggests they're remarkably willing to extend trust when that foundation exists. Studies show patients will even overlook their physician's conflicts of interest when they believe in the relationship.^31^

Medical ethicists often view insufficient “discounting” of medical advice to account for conflicts of interest as a cognitive failure requiring correction.^32^, ^33^ However, I think it reveals something about the nature of trust in medicine. Namely, public trust doesn't ultimately rest on p‐values or systematic methods; it resides in the relationship between a physician and their patient. In an era where artificial intelligence is rapidly encroaching on clinical practice and medicine grows increasingly commercialized, we must guard that relationship jealously. Trust in medicine can't be maintained with all the evidence in the world if patients fundamentally misunderstand it. For healthcare to be trusted, it requires physicians who can acknowledge uncertainty and the limitations inherent in medical interventions while demonstrating unwavering commitment to their patients’ well‐being.

## CONFLICT OF INTEREST STATEMENT

The author declares no conflict of interest.

## Data Availability

Data sharing is not applicable to this article as no datasets were generated or analysed during the current study.
